# High-resolution MRI synthesis using a data-driven framework with denoising diffusion probabilistic modeling

**DOI:** 10.1088/1361-6560/ad209c

**Published:** 2024-02-05

**Authors:** Chih-Wei Chang, Junbo Peng, Mojtaba Safari, Elahheh Salari, Shaoyan Pan, Justin Roper, Richard L J Qiu, Yuan Gao, Hui-Kuo Shu, Hui Mao, Xiaofeng Yang

**Affiliations:** 1 Department of Radiation Oncology and Winship Cancer Institute, Emory University, Atlanta, GA 30308, United States of America; 2 Department of Biomedical Informatics, Emory University, Atlanta, GA 30308, U nited States of America; 3 Department of Radiology and Imaging Sciences and Winship Cancer Institute, Emory University, Atlanta, GA 30308, United States of America

**Keywords:** MRI, diffusion model, high-resolution imaging, image synthesis, deep learning

## Abstract

*Objective*. High-resolution magnetic resonance imaging (MRI) can enhance lesion diagnosis, prognosis, and delineation. However, gradient power and hardware limitations prohibit recording thin slices or sub-1 mm resolution. Furthermore, long scan time is not clinically acceptable. Conventional high-resolution images generated using statistical or analytical methods include the limitation of capturing complex, high-dimensional image data with intricate patterns and structures. This study aims to harness cutting-edge diffusion probabilistic deep learning techniques to create a framework for generating high-resolution MRI from low-resolution counterparts, improving the uncertainty of denoising diffusion probabilistic models (DDPM). *Approach*. DDPM includes two processes. The forward process employs a Markov chain to systematically introduce Gaussian noise to low-resolution MRI images. In the reverse process, a U-Net model is trained to denoise the forward process images and produce high-resolution images conditioned on the features of their low-resolution counterparts. The proposed framework was demonstrated using T2-weighted MRI images from institutional prostate patients and brain patients collected in the Brain Tumor Segmentation Challenge 2020 (BraTS2020). *Main results*. For the prostate dataset, the bicubic interpolation model (Bicubic), conditional generative-adversarial network (CGAN), and our proposed DDPM framework improved the noise quality measure from low-resolution images by 4.4%, 5.7%, and 12.8%, respectively. Our method enhanced the signal-to-noise ratios by 11.7%, surpassing Bicubic (9.8%) and CGAN (8.1%). In the BraTS2020 dataset, the proposed framework and Bicubic enhanced peak signal-to-noise ratio from resolution-degraded images by 9.1% and 5.8%. The multi-scale structural similarity indexes were 0.970 ± 0.019, 0.968 ± 0.022, and 0.967 ± 0.023 for the proposed method, CGAN, and Bicubic, respectively. *Significance*. This study explores a deep learning-based diffusion probabilistic framework for improving MR image resolution. Such a framework can be used to improve clinical workflow by obtaining high-resolution images without penalty of the long scan time. Future investigation will likely focus on prospectively testing the efficacy of this framework with different clinical indications.

## 1. Introduction

Magnetic resonance imaging (MRI) offers high-contrast details for soft tissues, which benefit radiation treatment planning with correct material characterization for images with heterogenous structures (Chang *et al* 2023a). High-resolution MRI provides detailed anatomic structures that help the lesion detection and delineation (Pruessner *et al* 2000), enabling non-invasive and radiation-free assessment for lesion progression (Genovese *et al* 2019) and treatment management (Chang *et al* 2023b). However, high-resolution MRI scans require long image acquisition time to overcome the inherited low signal-to-noise ratio, especially when a large field of view (FOV) is used in body imaging (Plenge *et al* 2012, Shi *et al* 2015). Extending scan time is not only prohibitive when dealing with patients who cannot tolerate the long scan but also introduces more motion artifacts compromising the image quality (Pang *et al* 2016, Jang *et al* 2022, Chen *et al* 2023). More importantly, due to constraints from the fundamental physics applied in MRI data acquisition scheme and sequences to hardware limitations and safety concerns associated with RF power deposition to patients, resolution of clinical MRI sequences can be limited. Reducing image resolution can accelerate the acquisition time but with the risk of losing spatial details for potential lesion detection. This work aims to leverage a data-driven framework integrating state-of-the-art diffusion probabilistic deep learning to overcome those intrinsic limitations by synthesizing high-resolution MRI without sacrificing scan time and additional demands in the scanner hardware.

Upscaling image resolution is challenging since the approach is inherently ill-posed, leading to non-unique solutions. Conventional image high-resolution methods (Zhang *et al* 2008, Glasner *et al* 2009, Panda *et al* 2011) are typically based on Tikhonov regularization that requires an intricate prior to reconstructing local textures and edges from low-resolution images. Those methods (Rudin *et al* 1992) are usually built upon the assumption that a small local homogeneous region can be found in high-resolution images. However, this assumption is not valid when treatment sites include complex organs at risk with rich spatial details and fine structures. Such a bottom-up approach to image reconstruction relies on handcrafted features and struggles to capture complex, high-resolution image data with intricate patterns and structures. In contrast, data-driven approaches by deep learning have been proven as universal approximators (Hornik *et al* 1989, LeCun *et al* 2015) that can capture complex patterns and structures, allowing for highly realistic image generation. For instance, generative adversarial networks (GAN) have been investigated for reconstructing anatomical details from resolution-reduced MRI (Ledig *et al* 2017, Chen *et al* 2018, 2020, Cui *et al* 2022).

However, training methods based on GAN can be challenging due to the risk of training instability or model collapse, as observed in previous studies (He *et al* 2016, Huang *et al* 2017). It is crucial to exercise caution when applying these methods (Kodali *et al* 2017, Yi *et al* 2019, Gui *et al* 2023) in the context of medical image generation to ensure the quality of patient care. Medical imaging applications for radiotherapy have a unique requirement: synthetic high-resolution images need to preserve the features of images acquired from the treatment planning simulators for accurate target and organ contouring (Owrangi *et al* 2018, Otazo *et al* 2020). In addition, these simulators are calibrated to ensure the material details can be properly characterized from patient images to support radiation dose calculation for treatment planning (Chang *et al* 2020). Conserving the feature distribution is essential instead of seeking global minimum solutions as indicated by mean error-based metrics. A novel denoising diffusion probabilistic model (DDPM) (Jascha *et al* 2015, Ho *et al* 2020, Dhariwal and Nichol 2021) has demonstrated the capability to learn from data distributions and synthesize high-quality images. This learning approach empowers DDPM with outstanding proficiency in generating realistic nature images when compared to alternative deep learning methods (Nichol and Dhariwal 2021, Li *et al* 2022). DDPM is based on two distinct steps: (1) the forward diffusion process involves gradually introducing Gaussian noise to the input images until the image converges to an isotropic Gaussian distribution, (2) the reverse denoising process entails progressively eliminating noise from an initially noisy distribution until the genuine underlying data is revealed. This unique two-step approach empowers DDPM to capture intricate details and subtle variations, leveraging all the available, relevant, and adequately evaluated data (ARAED) (Chang and Dinh 2019). Consequently, DDPM can effectively leverage the entire data distribution while preserving crucial imaging features, a necessity in radiation treatment planning.

DDPM, representing a groundbreaking image synthesis model, generates high-quality images without common issues such as blurriness, mode collapse, or the lack of explicit likelihood estimation (Saharia *et al* 2023, Wu *et al* 2023). However, Lyu and Wang (2022) highlighted the inherent randomness associated with using DDPM for MRI image generation. Image uncertainty can propagate into radiation treatment planning, leading to an escalation of errors in dose calculation. This study aims to build a data-driven framework that incorporates a methodology for handling the stochastic nature of DDPM. The goal is to harness the adaptable and controllable structure of the newly devised DDPM to generate high-quality MRI images from lower-resolution inputs. Such a framework could significantly enhance the utility of MRI for several important tasks, including diagnosis, delineation, and the assessment of treatment response within the context of radiotherapy, potentially adding prognostic value. The main contributions of the current research can be encapsulated in two dimensions aimed at conducting a feasibility study for clinical implementation:

• The proposed framework introduces a stable method for generating high-resolution brain and prostate MRI images from low-resolution images, which applies to radiotherapy.
• The proposed framework is a valuable platform for investigating the conditions under which DDPM can consistently, efficiently, and accurately synthesize images, particularly in reconstructing high-resolution MRI images containing intricate local details.

## 2. Materials and methods

### 2.1. Patient data acquisition and data preprocessing

We used institutional and open-access MRI datasets to investigate the proposed framework for high-resolution MRI synthesis. The institutional MR images (Zhou *et al* 2022) were acquired from 36 patients with cT1-3bN0 prostate cancer. The ‘cT1-3bN0 prostate cancer’ indicates that the clinical assessment of the primary tumor falls into one of the categories cT1 to cT3, and no cancer cells were found in lymph node biopsies (bN0). The diagnosis images were acquired using a T2-weighted turbo spin echo sequence from a Siemens MAGNETOM Aera 1.5T scanner with a 1 mm slice thickness. The total prostate MRI dataset included 2696 image slices with a dimension of 256 × 256 pixels with a FOV of 250 mm and a mean slice number of 75 for each patient. The brain MRI images were obtained from the Brain Tumor Segmentation Challenge 2020 (BraTS2020) (Menze *et al* 2015, Bakas *et al* 2017, 2018). We used the T2-weighted fluid attenuated inversion recovery (T2-FLAIR) from patients in the BraTS2020 dataset, including 23 897 image slices with a dimension of 240 × 240 pixels with slice thicknesses of 2–6 mm, and FOV of 200–240 mm.

The raw data from both image databases served as the high-resolution MRI datasets, and the images from BraTS2020 were padded by zero to reach the dimension of 256 × 256 pixels. The low-resolution MR images were down-sampled based on the high-resolution images from 256 × 256 pixels to 86 × 128 pixels (6x resolution-downgraded). We randomly sampled six patients from prostate MR image sets as testing data such that the dataset included 2256 and 440 slices for training and testing. For brain T2-FLAIR images, 12 patients were randomly sampled from BraTS2020 such that the brain dataset contained 22 884 and 1013 slices for training and testing.

### 2.2. Data-driven framework for high-resolution MRI synthesis

**Figure 1.**
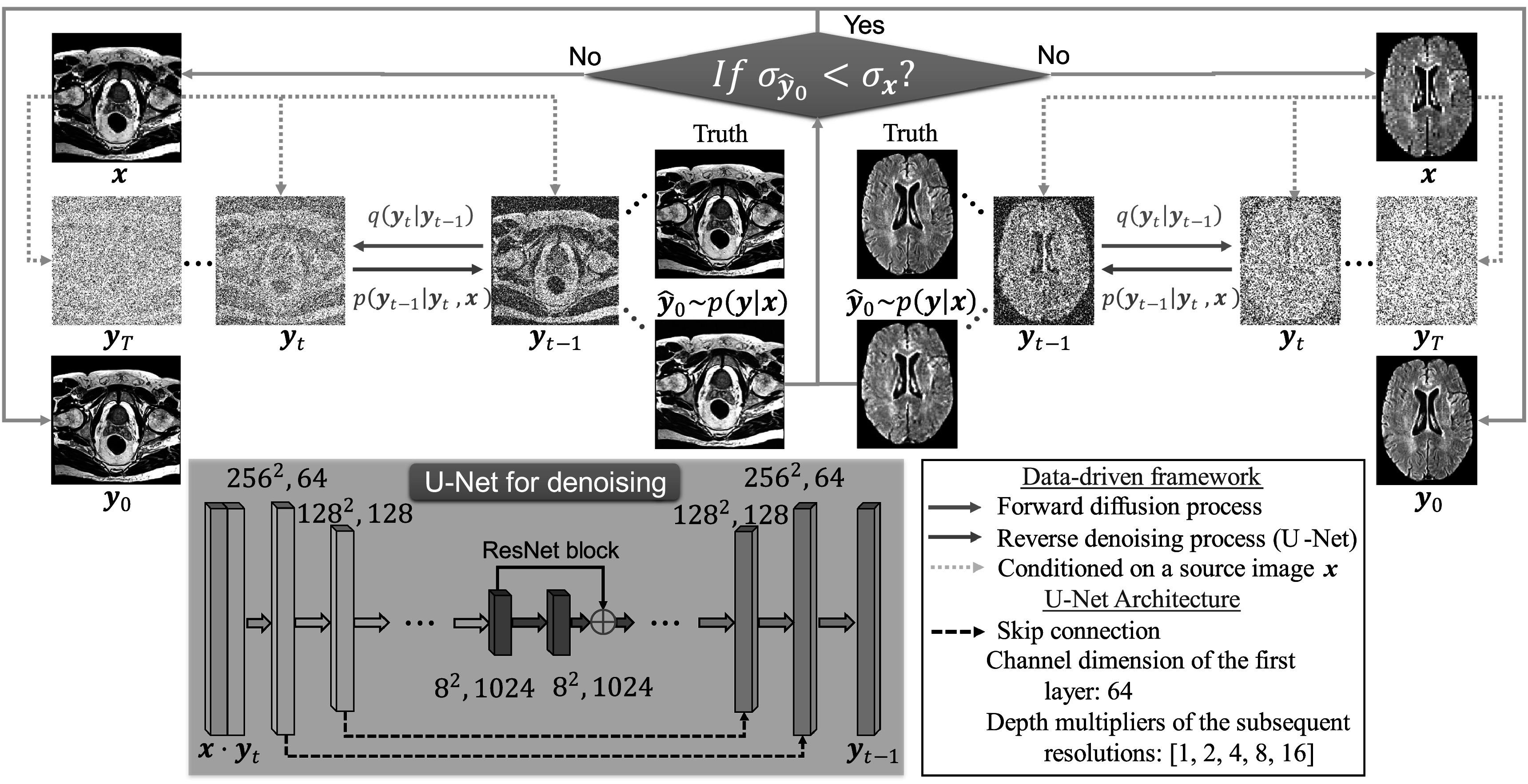
Overview of the proposed data-driven framework for high-resolution MRI synthesis.

The proposed data-driven framework integrates a PyTorch-based DDPM (Saharia *et al* 2023) and a patch-based image noise estimation method (Liu *et al* 2012, 2013) to robustly synthesize high-quality images. Figure 1 depicts the proposed framework, including the low-resolution brain and prostate MRI images as inputs (***x***) and the high-resolution MR images as the targets (Truth). The framework includes two processes: (a) the DDPM process is primary for high-resolution image synthesis, and blue and red arrows denote the process, (b) the image noise estimation process is designed to ensure the applicability of generated images, and gray arrows denote this process. The proposed framework iterates the two processes until high-quality images are generated for radiotherapy applications.

The DDPM model comprises two distinct steps, the forward process (*q*) and the reverse process (*p*), denoted by the blue and red arrows in figure 1. The forward diffusion process (blue workflow) uses the Markov chain to systematically add Gaussian noise to the target images (Truth) through different time steps (*t*). This forward process continues until the image converges to an isotropic Gaussian distribution (***y***_*T*_). For the reverse process (red workflow), the goal is to remove the Gaussian noise from the image (***y***_*T*_) with guidance from the low-resolution images (***x***) to reconstruct high-resolution images (${\hat{{\boldsymbol{y}}}}_{0}$). This process is achieved by training U-Net models (Brock *et al* 2018, Song *et al* 2020) to learn how to remove the noise from the image (***y***_*t*_) at different time steps (*t*). This supervised training is possible since the added Gaussian noises are known from the forward process.

Due to the stoichiometric nature of DDPM, the generated image quality can be unacceptable for insufficient sampling. To define the acceptable condition, we hypothesize that the estimated noise level (ENL) of synthetic MR images (${\sigma }_{{\hat{{\boldsymbol{y}}}}_{0}}$) should be less than the noise level of low-resolution images (${\sigma }_{{\boldsymbol{x}}}$) to conserve the image features from actual acquisition. We implement a decision block (blue diamond box) in figure 1 to evaluate the noise level of synthetic MRI images. If the synthetic image fails to meet the hypothesis, the framework will reject the image, and a new image will be regenerated from the DDPM processes.

The proposed framework in figure 1 will iterate between the DDPM and noise estimation processes until acceptable images are generated for radiotherapy. More details of the framework implementation are given in the following sub-sections: section 2.2.1 gives the model parameters of DDPM, section 2.2.2 and section 2.2.3 show the details of the forward process and reverse process of DDPM, section 2.2.4 introduces how to implement the proposed framework with DDPM and the noise estimation process.

#### 2.2.1. Denoising diffusion probabilistic model

We implemented the state-of-the-art super-resolution DDPM (SR3-DDPM) (Saharia *et al* 2023) to translate a 2D low-resolution MR image into a high-resolution image. Figure 1 shows that this technique utilizes a conditional diffusion-based process to transform isotropic Gaussian noise samples (***y***_T_) into a high-resolution image (${\hat{{\boldsymbol{y}}}}_{0}$), conditioned on the same image at the low-resolution level (***x***). The diffusion process (*q*) assumes that by adding a small amount of Gaussian noise *ε* ∼ *N(*0*, **I**)* to the target image (Truth) over *t* timesteps until the image contains a purely Gaussian noise (***y***_*T*_) as *t* is sufficiently large. We used *t* equal to 5000 in this work, and the added Gaussian noise to the high-resolution image generated a sequence of noisy images with increasing noise level: $y\in [{y}_{1},{y}_{2},\ldots ,{y}_{T}],$ where *T* equal to 5000 is the pre-determined maximum timestep. For the reverse process (*p*), we train a U-Net model (Brock *et al* 2018, Song *et al* 2020) conditioned on the low-resolution images (***x***) with a loss function formulated based on Kullback-Leibler divergence to maximize the likelihood of the target images (Truth) and synthetic high-resolution images (${\hat{{\boldsymbol{y}}}}_{0}$). The U-Net takes the low-resolution images as inputs (***x***), which will be up-sampled using bicubic interpolation to the dimensions of 256 × 256 pixels. Then ***x*** will be concatenated with the noisy image (*y*_*t*_) to output the denoised image (*y*_*t−*1_). We implemented the U-Net with 64 channels for the first layer and the depth multipliers were 1, 2, 4, 8, and 16 applied to the subsequent resolutions. The network also included a ResNet block given in figure 1.

#### 2.2.2. Forward gaussian diffusion process

The forward diffusion process (Jascha *et al* 2015, Ho *et al* 2020) is designed to follow the Markov process to gradually add Gaussian noise to the target image (high-resolution MRI) step by step from *y*_*t−1*_ to *y*_*t*_, as shown in figure 1. Equation (1) shows the step-wised generation process of embedding Gaussian noises to the target at timestep *t* where *α* is the hyper-parameter to determine the spread of noise added in each step, and its values are subject to the range of (0,1). Equation (2) gives the complete generation process of Gaussian noise embedding from the target images, where *T* denotes the maximum timestep. Equation (2) can be further simplified by directly correlating *y*_*t*_ to *y*_*0*_ as given by equation (3) where ${\gamma }_{t}={\prod }_{i=1}^{t}{\alpha }_{i}.$

\begin{eqnarray*}q\left({y}_{t},|,{y}_{t-1}\right)={\mathrm{\unicode{x1D4A9}}}\left({y}_{t}{\mathrm{|}}\sqrt{{\alpha }_{t}}{y}_{t-1},(1-{\alpha }_{t})I\right)\end{eqnarray*}

\begin{eqnarray*}q\left({y}_{1:T},|,{y}_{0}\right)=\displaystyle \prod _{t=1}^{T}q\left({y}_{t},|,{y}_{t-1}\right)\end{eqnarray*}

\begin{eqnarray*}q\left({y}_{t},|,{y}_{0}\right)={\mathrm{\unicode{x1D4A9}}}\left({y}_{t}{\mathrm{|}}\sqrt{{\gamma }_{t}}{y}_{0},(1-{\gamma }_{t})I\right)\end{eqnarray*}

Equation (3) indicates that the forward process of noisy image (*y*_*t*_) at arbitrary timestep *t* can be associated with the target image (*y*_*0*_). To obtain the noisy image (*y*_*t*_) from *y*_*0*_ in figure 1, equation (4) shows that *y*_*t*_ can be sampled from a normal distribution based on equation (3) where *ε* ∼${\mathrm{\unicode{x1D4A9}}}$(0*, I)*.

\begin{eqnarray*}{y}_{t}=\sqrt{1-{\gamma }_{t}}\varepsilon +\sqrt{{\gamma }_{t}}{y}_{0}\end{eqnarray*}

Furthermore, Ho *et al* (2020) have demonstrated that the probability distribution of *y*_*t−1*_ given (*y*_*0*_, *y*_*t*_) can be presented as equation (5) with the mean and variance given in equations (6) and (7).

\begin{eqnarray*}q\left({y}_{t-1},|,{y}_{0},{y}_{t}\right){\mathscr{=}}{\mathscr{N}}\left({y}_{t-1}{\mathrm{|}}\mu ,{\sigma }^{2}I\right)\end{eqnarray*}

\begin{eqnarray*}\mu ={\left(1-{\gamma }_{t}\right)}^{-1}\left[\sqrt{{\gamma }_{t-1}}\left(1-{\alpha }_{t}\right){y}_{0}+\sqrt{{\alpha }_{t}}(1-{\gamma }_{t-1}){y}_{t}\right]\end{eqnarray*}

\begin{eqnarray*}{\sigma }^{2}={\left(1-{\gamma }_{t}\right)}^{-1}(1-{\gamma }_{t-1})\left(1-{\alpha }_{t}\right).\end{eqnarray*}

#### 2.2.3. Reverse denoising process

The reverse denoising process aims to remove the Gaussian noise from the forward diffusion process. This approach is a reverse Markovian process starting from the noisy image (*y*_*T*_). Figure 1 shows that this inverse approach takes a small step to restore the image structure from *y*_*t*_ to *y*_*t−1*_ to gradually remove the noise from images, and the process will be recursively executed until a corresponding high-resolution image (${\hat{y}}_{0}$) is generated. A U-Net model (*f*_*θ*_) (Ronneberger *et al* 2015) was trained to denoise images from the forward process by estimating *ε* in equation (4) using the low-resolution image (*x*) and the noisy image (*y*_*t*_) as model inputs. Equation (8) shows the objective function for training where *T* is the total timesteps.

\begin{eqnarray*}\mathop{{\mathrm{argmin}}\,}\limits_{\theta }L=\frac{1}{T}\displaystyle \sum _{t=1}^{T}{\unicode{x02016}{f}_{\theta }(x,{y}_{t},{\gamma }_{t})-\varepsilon \unicode{x02016}}_{2}^{2}.\end{eqnarray*}

The denoising process is achieved by estimating the probability distribution of *p*_*θ*_(*y*_*t−1*_|*y*_*t*_,*x*) with the condition on the low-resolution image (*x*) as the prior for the inference. Equation (9) shows the posterior distribution of *p*_*θ*_ where *μ*_*θ*_ is given by equation (10).

\begin{eqnarray*}{p}_{\theta }({y}_{t-1}{\mathrm{\unicode{x02502}}}{y}_{t},x{\mathscr{)}}{\mathscr{=}}{\mathscr{N}}\left({y}_{t-1}{\mathrm{|}}{\mu }_{\theta }(x,{y}_{t},{\gamma }_{t}),{\sigma }^{2}I\right)\end{eqnarray*}

\begin{eqnarray*}{\mu }_{\theta }(x,{y}_{t},{\gamma }_{t})=\frac{1}{\sqrt{{\alpha }_{t}}}\left[{y}_{t}-\frac{1-{\alpha }_{t}}{\sqrt{{1-\gamma }_{t}}}{f}_{\theta }(x,{y}_{t},{\gamma }_{t})\right].\end{eqnarray*}

Finally, we define a constant variance (1−*α*_*t*_) for *p*_*θ*_(*y*_*t−1*_|*y*_*t*_,*x*) that allows us to use equation (11) to iteratively sample *y*_*t−1*_ from a normal distribution, *ε* ∼${\unicode{x1D4A9}}$(0*, I)*, based on equation (9). By taking equation (10) into equations (11), (12) shows the ultimate denoising model that can synthesize high-resolution images ${\hat{y}}_{0}$ from Gaussian noisy sample (*y*_*T*_), which are conditioned on low-resolution images (*x*) in figure 1.

\begin{eqnarray*}{y}_{t-1}=\sqrt{{1-\alpha }_{t}}{\varepsilon }_{t}+{\mu }_{\theta }(x,{y}_{t},{\gamma }_{t})\end{eqnarray*}

\begin{eqnarray*}{y}_{t-1}=\sqrt{{1-\alpha }_{t}}{\varepsilon }_{t}+\frac{1}{\sqrt{{\alpha }_{t}}}\left[{y}_{t}-\frac{1-{\alpha }_{t}}{\sqrt{{1-\gamma }_{t}}}{f}_{\theta }(x,{y}_{t},{\gamma }_{t})\right].\end{eqnarray*}

#### 2.2.4. Implementation of the data-driven framework for high-resolution MR image synthesis

To ensure the robustness of the proposed framework in figure 1, we hypothesized that the synthetic high-resolution MR image should achieve comparable image noise levels. The proposed framework integrated an image noise estimation method to ensure the convergence of generated MR images. In this work, we implemented a patch-based noise estimation method (Liu *et al* 2012) using principal component analysis to ensure the performance of high-resolution MRI syntheses.

Liu *et al* (2013) proposed a patch-based noise estimation method for a single image. Equation (13) gives the ENL for an image where *y*, Σ, and *λ*_min_ denote the image, the covariance matrix of the image, and the minimum eigenvalue of the covariance matrix. Equation (14) shows the equation for deriving the covariance matrix (Σ) given an image (*y*) where *M* and *z*_*i*_ present the number of patches and the ith image patch with the dimension of 7 × 7 pixels in this work. Ultimately, Algorithm 1 shows the framework implementation for inferring high-resolution MR images from low-resolution MR images.

\begin{eqnarray*}{\sigma }^{2}={\lambda }_{\min }\left({{\mathrm{\Sigma }}}_{y}\right)\end{eqnarray*}

\begin{eqnarray*}{{\mathrm{\Sigma }}}_{y}=\frac{1}{M}\displaystyle \sum _{i=1}^{M}{z}_{i}{z}_{i}^{T}.\end{eqnarray*}

**Table 2.** Quantitative results achieved by the proposed data-driven framework for high-resolution brain MRI synthesis using the brain MRI BraTS2020 dataset. Multiple evaluation metrics are used to compare the ground truth to low-resolution (LR) images, Bicubic images, and synthetic images by the CGAN and the proposed data-driven framework. Arrows specify the lower and higher values of a given quantitative metric represent better performance.

		MAE [↓]	NQM (dB) [↑]	PSNR (dB) [↑]	MSSIM [↑]	LPIPS [↓]
LR	Mean ± SD	21.03 ± 7.02	16.04 ± 3.12	26.25 ± 3.42	0.964 ± 0.026	0.095 ± 0.015
Bicubic	Mean ± SD	18.55 ± 6.39	16.18 ± 3.07	27.77 ± 3.69	0.967 ± 0.023	0.132 ± 0.017
	Enhancement from LR	11.8%	0.9%	5.8%	0.3%	−39.1%
CGAN	Mean ± SD	**17.71** ± 5.84	16.64 ± 3.37	25.79 ± 3.31	0.968 ± 0.022	0.075 ± 0.016
	Enhancement from LR	**15.8%**	3.7%	−1.8%	0.4%	20.9%
Proposed	Mean ± SD	22.40 ± 7.33	**16.70** ± 2.81	**28.63** ± 2.98	**0.970** ± 0.019	**0.057** ± 0.009
	Enhancement from LR	−6.5%	**4.1%**	**9.1%**	**0.6%**	**40.1%**
*p*-value	Proposed versus LR	<0.01	<0.01	<0.01	<0.01	<0.01
	Proposed versus Bicubic	<0.01	< 0.01	< 0.01	< 0.01	<0.01
	Proposed versus CGAN	<0.01	**0.672**	<0.01	<0.01	<0.01

### 2.3. Framework performance quantification

To assess the performance of the proposed diffusion model, we have implemented two benchmark models: the clinical bicubic interpolation model (Keys 1981) (referred to as ‘Bicubic’) and a conditional generative adversarial network (CGAN) (Isola *et al* 2017). Our evaluation of the framework’s reliability involved utilizing a MATLAB package (Gaubatz 2014 [Bibr pmbad209cbib19]) to analyze the synthetic MR images. This comprehensive assessment included the computation of various metrics, namely the mean absolute error (MAE) (Chang *et al* 2022a), peak signal-to-noise ratio (PSNR) (Faragallah *et al* 2021), noise quality measure (NQM) (Damera-Venkata *et al* 2000), multi-scale structural similarity (MSSIM) (Wang *et al* 2003), and learned perceptual image patch similarity (LPIPS) (Zhang *et al* 2018).

The MAE metric served as a means to measure the average pixel-wise discrepancies between the generated images and their corresponding ground truth images. It allowed us to quantify the overall dissimilarity between these image pairs. Furthermore, to gauge the quality of the synthetic images, we used the PSNR metric. PSNR was employed to quantify the level of fidelity exhibited by the generated images compared to their ground truth images.

For evaluating visual quality and the preservation of structural details in relation to the reference image, we employed the NQM metric. NQM took into account factors such as distance from the reference image, image dimensions, and spatial frequencies. While the conventional SSIM index, as introduced by Zhou *et al* (2004), is a single-scale approach that may not always accurately evaluate image quality based on different viewing conditions, we adopted the MSSIM metric. MSSIM allowed us to assess the generated images with consideration for various viewing conditions, including factors such as display resolution and viewing distance.

LPIPS serves as a metric for assessing the perceptual similarity between two images. LPIPS is derived from a model trained on a labeled dataset, where human judgments determine perceptual similarity. A higher LPIPS value indicates greater dissimilarity or distance, whereas a lower metric suggests a higher degree of similarity between the images.

We employed a set of diverse metrics to comprehensively evaluate the trustworthiness of the framework’s results when applied to synthetic MR images. These metrics collectively provided a robust assessment of the synthetic image quality, structural preservation, and visual fidelity, accommodating various viewing conditions.

## 3. Results

### 3.1. Comparisons of estimated noise level between the proposed data-driven framework and a denoising diffusion probabilistic model

**Figure 2.**
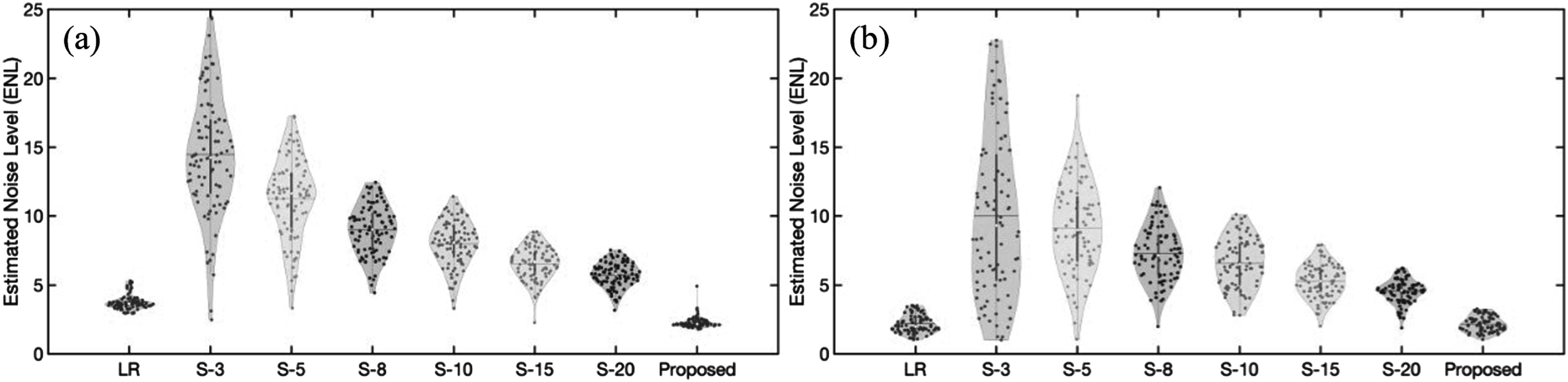
Comparisons of estimated noise level (ENL) distributions for all patients’ MR images from (a) the institutional prostate and (b) BraTS2020 datasets including low-resolution (LR) images, synthetic MRI by the proposed framework, and averaged MR images by running a denoising diffusion probabilistic model (DDPM) multiple times to acquire different sample sizes (S-3 ∼ S-20).

The proposed data-driven framework has the capability to determine the final synthetic high-resolution MR images based on the ENL. In figure 2, we present comparisons of the estimated image noises between low-resolution MRI scans and the images generated using our proposed method and DDPM techniques. This analysis uses complete testing patient image sets from institutional prostate and BraTS2020 datasets.

Given the probabilistic nature of the denoising processes involved in DDPM, the DDPM-generated images were obtained by averaging results from different sample sizes, achieved by running the prediction model multiple times. For instance, S-3 in figure 2 represents the generated images obtained by averaging the outputs of the DDPM model over three runs. As the sample sizes increase, ENL’s mean and variance values decrease for DDPM-generated images. Figure 2 illustrates that our proposed framework consistently attains the smallest mean ENL values when applied to prostate and brain datasets, outperforming the DDPM approach.

**Figure 3.**
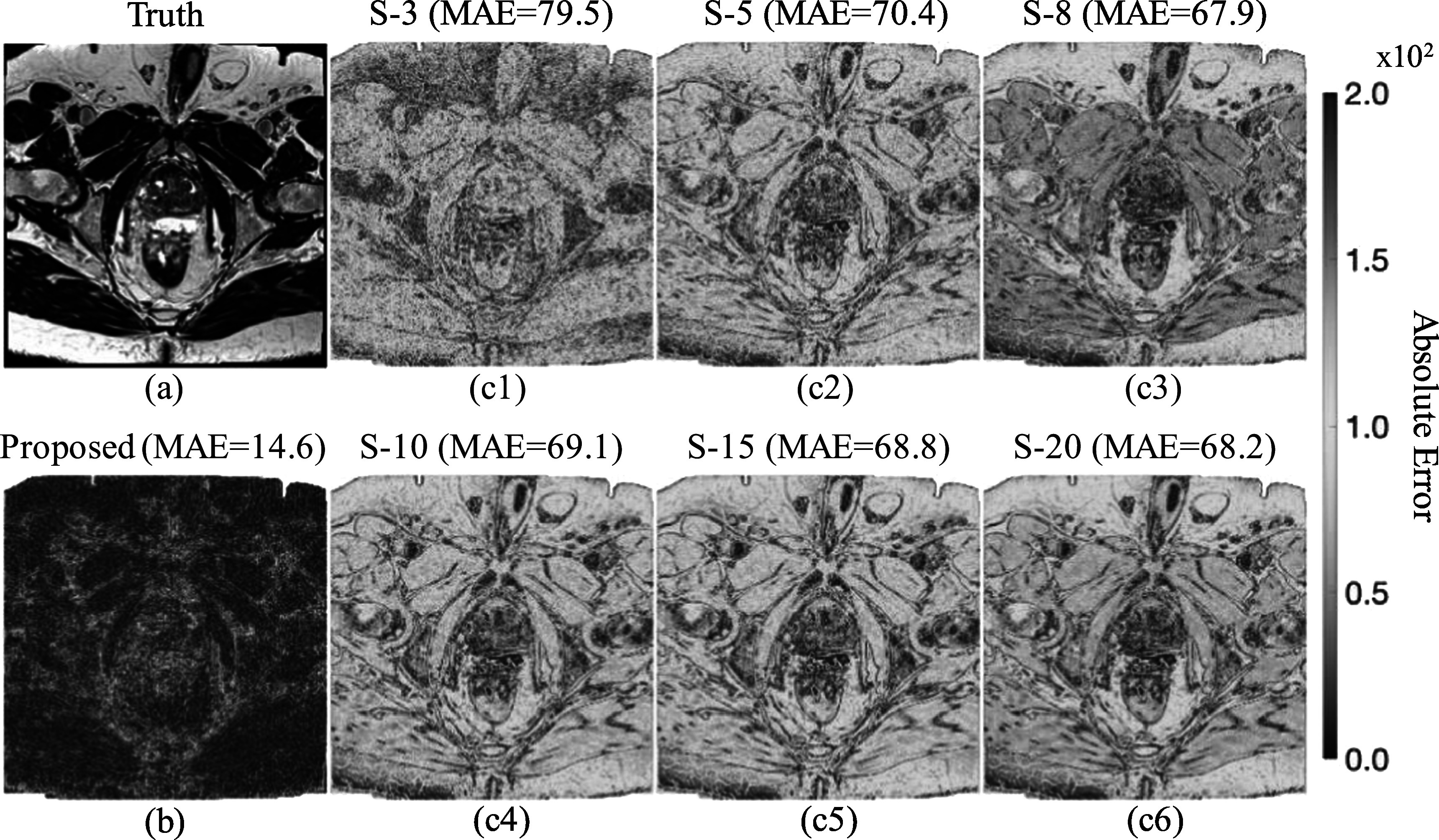
Comparisons of absolute error maps between (a) the ground truth and synthetic prostate MR images by (b) the proposed framework and (c1–c6) by running a denoising diffusion probabilistic model (DDPM) multiple times to acquire averaged MR images from different sample sizes (S-3 ∼ S-20). MAE stands for the mean absolute error.

**Figure 4.**
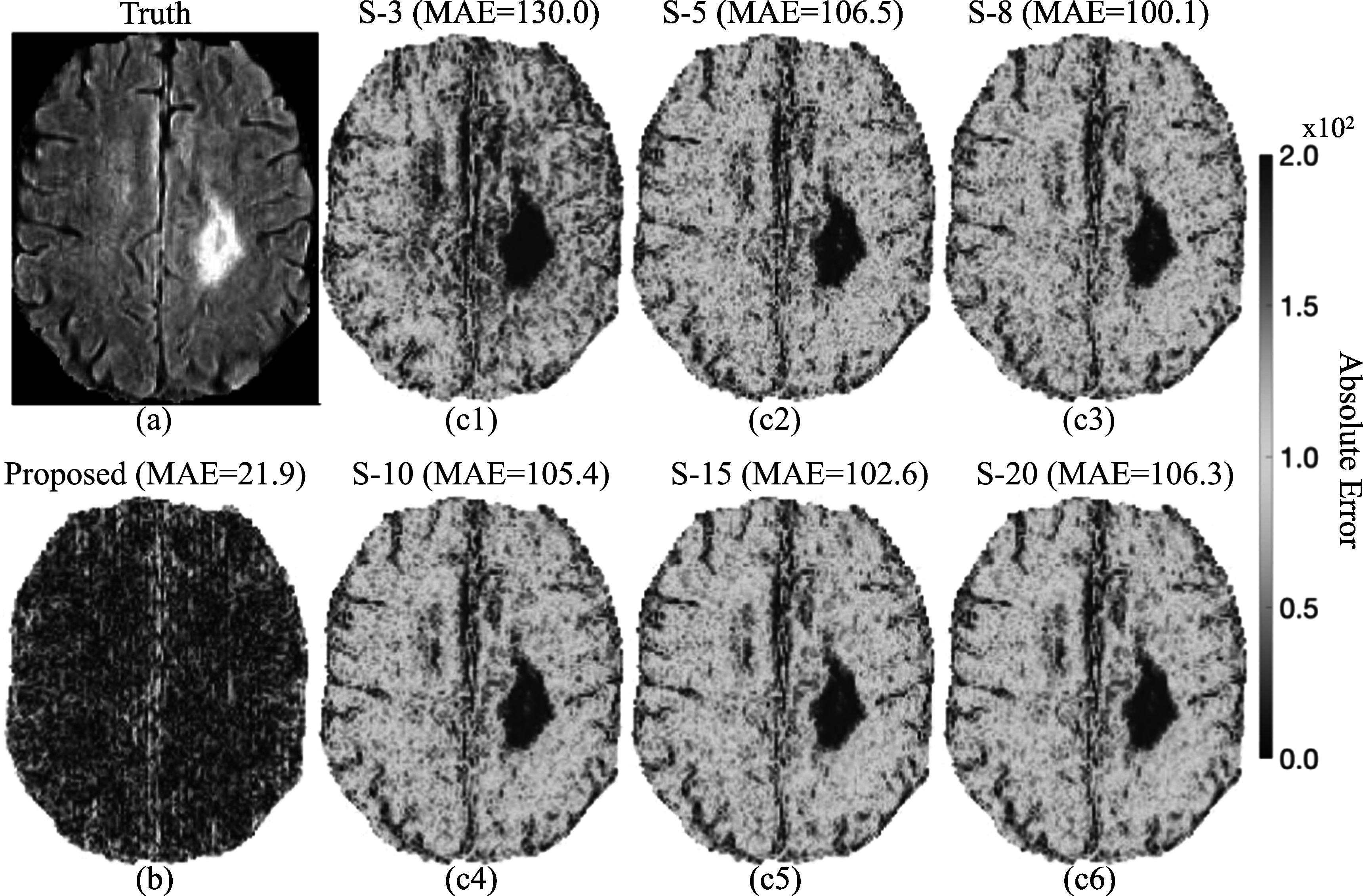
Comparisons of absolute error maps between (a) the ground truth and synthetic brain MR images by (b) the proposed framework and (c1–c6) by running a denoising diffusion probabilistic model (DDPM) multiple times to acquire averaged MR images from different sample sizes (S-3 ∼ S-20). MAE stands for the mean absolute error.

Figure 3 displays the absolute difference maps between the synthetic high-resolution MR images and the ground truth. Figure 3(b) shows that the proposed data-driven framework surpasses other methods for high-resolution image synthesis, achieving the best MAE value of 14.6. The absolute error map (AE map) demonstrates a strong consistency between the generated high-resolution images and the ground truth, as shown in figure 3(a). Regarding the DDPM model, figures 3(c1)–(c6) illustrate a decrease in MAE values as the sample sizes used for image averaging increase. However, even when running DDPM 20 times, the generated images still exhibit a significant MAE difference of 68.2 compared to the ground truth. Figure 4 shows the absolute difference maps between the synthetic brain MR images and the ground truth. The proposed method achieved the optimal MAE value of 21.9. Figures 4(c1)–(c6) depict that the MAE decreases as the increase of the sample sizes for averaging.

### 3.2. Synthesis of high-resolution images using the institutional prostate dataset

We conducted a performance comparison of our proposed framework for generating high-resolution MRI against other methods, specifically Bicubic and CGAN, in order to illustrate its impact. Image generation times for CGAN and the proposed framework were 1.2 ± 0.7 and 21.0 ± 0.6 s/slice. As demonstrated in table 1, our proposed method yields the most favorable results when evaluated using metrics such as NQM and PSNR, scoring 18.9 ± 1.6 dB and 25.9 ± 1.7 dB, respectively. These scores represent significant improvements of 12.8% and 11.7% over the low-resolution images.

**Algorithm 1.** High-resolution MRI synthesis using the proposed data-driven framework as given in figure 1.

Algorithm 1 Image inference with total timesteps *T*
**Input:** Low-resolution MRI images (*x*)
**Output:** Synthetic high-resolution MRI images (${\hat{y}}_{0}$)
1: Estimate ${\sigma }_{x}$ equation (14)
2: **repeat**
3: Sample *y*_*T*_ from *N(0,I)*
4: **for** *t* = *T*, …, 1 **do**
5: *ε*_*t*_ ∼ *N(0,I)* **if** *t* > 1 **else** *ε*_*t*_ = 0
6: ${y}_{t-1}=\sqrt{{1-\alpha }_{t}}{\varepsilon }_{t}+\frac{1}{\sqrt{{\alpha }_{t}}}\left[{y}_{t}-\frac{1-{\alpha }_{t}}{\sqrt{{1-\gamma }_{t}}}{f}_{\theta }(x,{y}_{t},{\gamma }_{t})\right]$
7: **until** ${\sigma }_{{\hat{y}}_{0}}\leqslant {\sigma }_{x}$

Comparing the MAE results to those obtained with resolution-degraded images, the Bicubic and our proposed framework enhance the MAE value by 14.7% and 12.5%, respectively. Regarding MSSIM analysis, the Bicubic shows a marginal improvement of only 1% in structural similarity value compared to the low-resolution images. In contrast, the deep learning-based methods achieve a minimum improvement of 1.6% over the low-resolution images.

**Figure 5.**
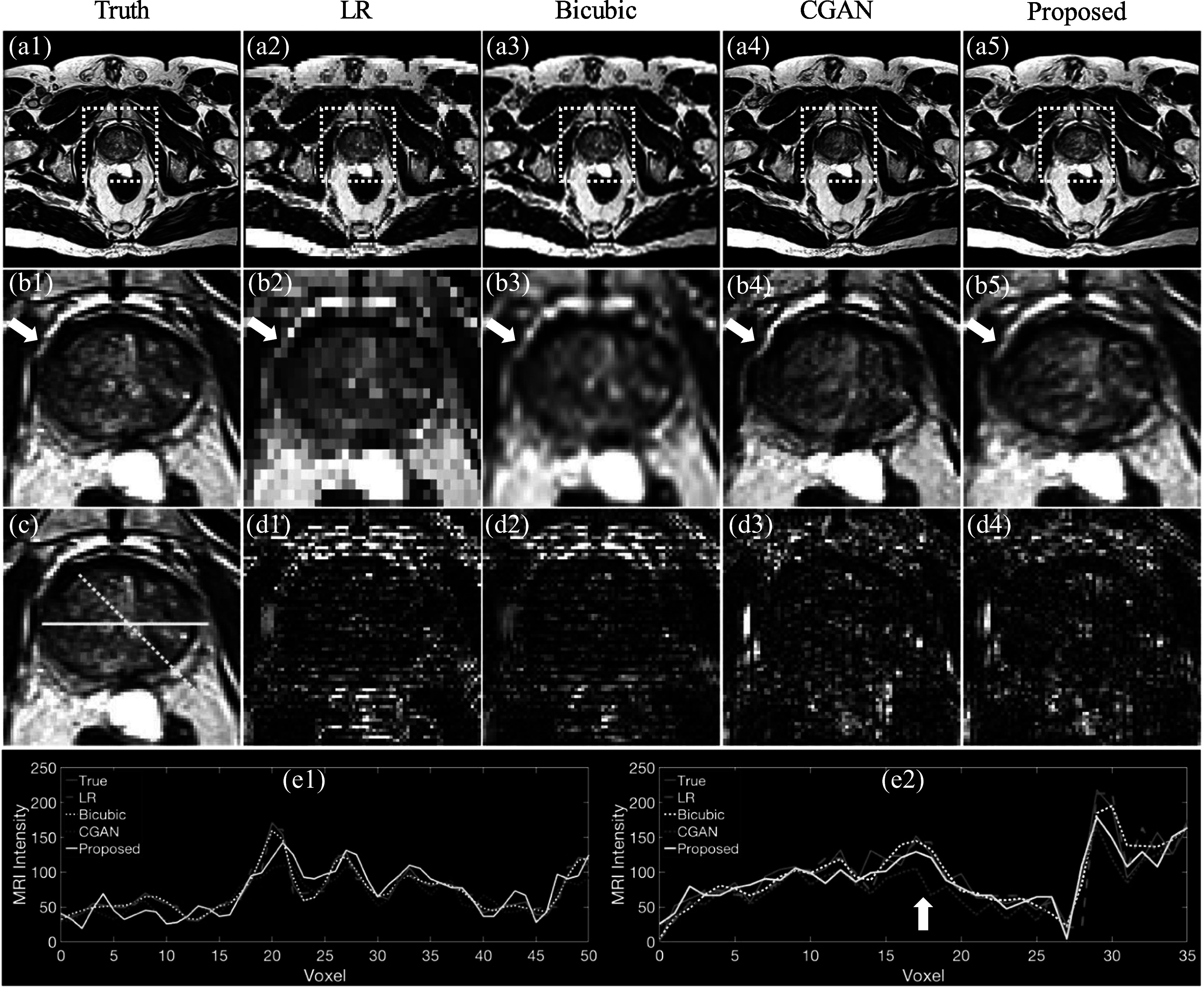
Using the proposed probabilistic diffusion deep learning model to increase image quality for low-resolution (LR) prostate MR images. MR images of (a1) ground truth, (a2) LR, (a3) Bicubic, (a4) conditional GAN (CGAN), and (a5) proposed diffusion model. (b1)–(b5) Zoom-in plots for the yellow dotted box in (a1)–(a5). (c) The yellow solid line and dashed line show the line profiles given in (e1)–(e2). (d1)–(d4) give each method’s absolute error between ground truth and images. (e1)–(e2) shows the horizontal and oblique profiles as indicated by (c). The window levels are [0, 200] and [0, 100] for (a1)–(c) and (d1)–(d4). The white arrows indicate the edge deformation caused by CGAN.

Figure 5 shows high-resolution prostate MR images generated using various methods. Specifically, figures 5(b1)–(b4) provide detailed comparisons of each image, focusing on the prostate region. Notably, the deep learning-based methods exhibit superior image resolution compared to the images generated through Bicubic interpolation. Figure 5(b4) reveals that CGAN produces prostate MR images with a distorted prostate boundary, as the white arrow indicates, whereas the proposed method accurately preserves the boundary.

Figures 5(d1)–(d2) illustrate that low-resolution (LR) and Bicubic images result in more significant absolute errors in the anterior prostate region, in contrast to CGAN and the proposed method. Figure 5(e2) presents the oblique line profile, as depicted in figure 5(c), revealing that CGAN consistently underestimates MRI intensities in the central prostate region. At the same time, the proposed method generates a profile that aligns consistently with the ground truth.

### 3.3. Synthesis of high-resolution images using the BraTS2020 dataset

To broaden the proposed framework’s application scope, we employed brain T2-FLAIR images from the publicly accessible BraTS2020 dataset. Image generation times for CGAN and the proposed framework were 1.4 ± 1.3 and 20.3 ± 0.4 s/slice. Table 2 presents quantitative comparisons between our proposed methods and alternative approaches, including Bicubic and CGAN. The proposed framework exhibited superior performance when assessed through various evaluation metrics, including NQM, PSNR, and MSSIM, yielding 16.7 ± 2.8 dB, 28.6 ± 3.0 dB, and 0.97 ± 0.02, respectively. These values represent a notable improvement of 4.1%, 9.1%, and 0.6% compared to the low-resolution images. The proposed method achieves the optimal perceptual similarity based on the LPIPS metric with the value of 0.057 ± 0.009. The CGAN method achieved the highest MAE value of 17.7 ± 5.8, signifying a 15.8% enhancement over the low-resolution images. It is worth noting that both deep learning-based methods provided improved visual quality, as indicated by MSSIM and LPIPS, in contrast to Bicubic.

**Table 1.** Quantitative results achieved by the proposed data-driven framework for high-resolution prostate MRI synthesis using the institutional dataset. Multiple evaluation metrics are used to compare the ground truth to low-resolution (LR) images, Bicubic images, and synthetic images by the CGAN and the proposed data-driven framework.

		MAE [↓]	NQM (dB) [↑]	PSNR (dB) [↑]	MSSIM [↑]	LPIPS [↓]
LR	Mean ± SD	16.94 ± 2.16	16.74 ± 1.75	23.10 ± 2.03	0.945 ± 0.013	0.283 ± 0.024
Bicubic	Mean ± SD	**14.46** ± 1.83	17.48 ± 1.86	25.32 ± 2.18	0.954 ± 0.011	0.316 ± 0.029
	Enhancement from LR	**14.7%**	4.4%	9.8%	1.0%	−11.4%
CGAN	Mean ± SD	14.52 ± 1.41	17.70 ± 1.87	25.50 ± 1.88	**0.961** ± 0.010	**0.071** ± 0.006
	Enhancement from LR	14.3%	5.7%	8.1%	**1.7%**	**74.9%**
Proposed	Mean ± SD	14.83 ± 1.54	**18.88** ± 1.58	**25.93** ± 1.72	0.960 ± 0.008	0.077 ± 0.006
	Enhancement from LR	12.5%	**12.8%**	**11.7%**	1.6%	72.8%
*p*-value	Proposed versus LR	<0.01	<0.01	<0.01	<0.01	<0.01
	Proposed versus Bicubic	<0.01	<0.01	<0.01	<0.01	<0.01
	Proposed versus CGAN	<0.01	<0.01	<0.01	**0.02**	**0.12**

**Figure 6.**
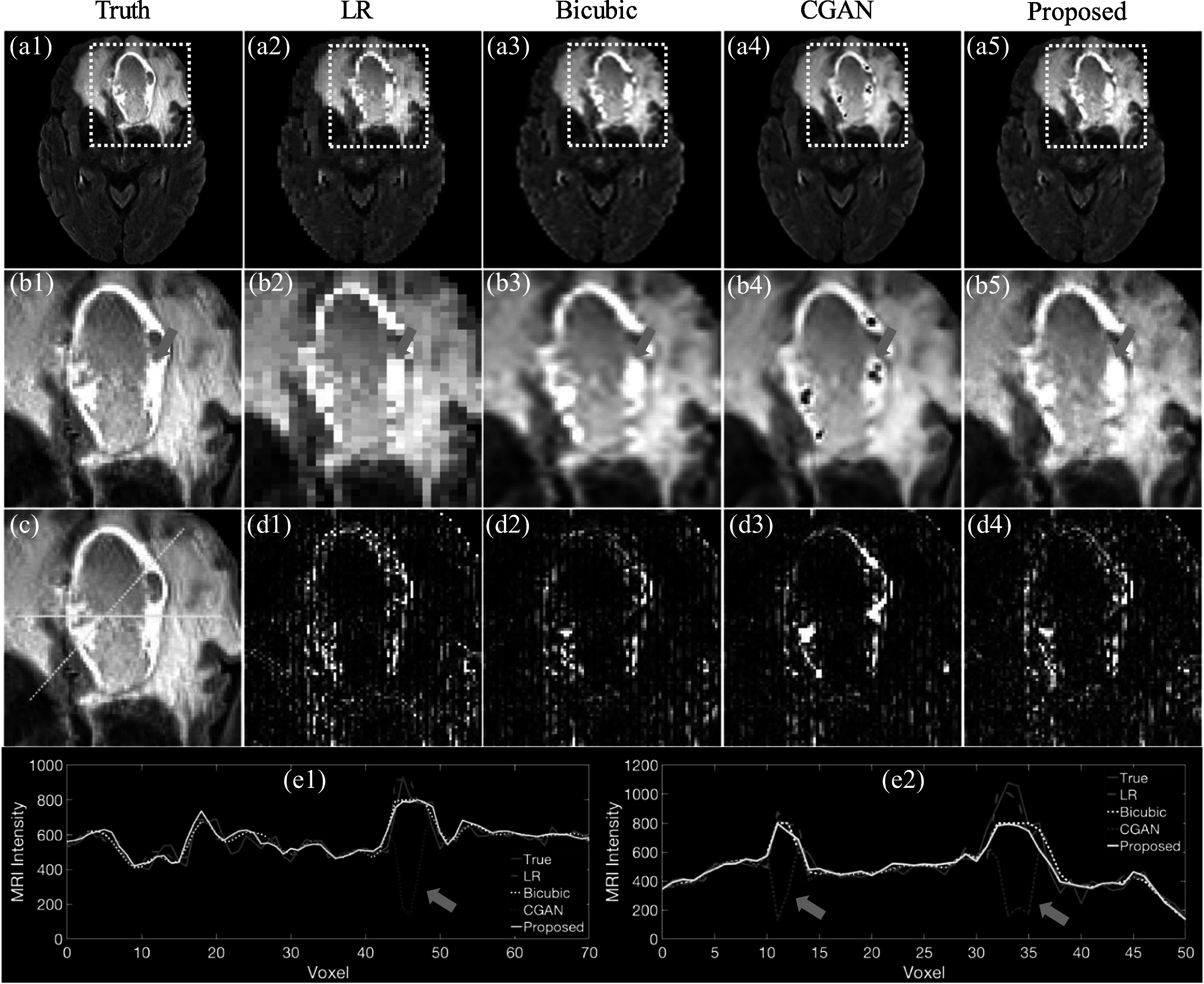
Using the proposed probabilistic diffusion deep learning model to increase image quality for low-resolution (LR) brain MR images. MR images of (a1) ground truth, (a2) LR, (a3) Bicubic, (a4) conditional GAN (CGAN), and (a5) proposed diffusion model. (b1)–(b5) Zoom-in plots for the yellow dotted box in (a1)–(a5). (c) The yellow solid line and dashed line show the line profiles given in (e1)–(e2). (d1)–(d4) Give each method’s absolute error between ground truth and images. (e1)–(e2) Shows the horizontal and oblique profiles as indicated by (c). The window levels are [0, 700] and [0, 200] for (a1)–(c) and (d1)–(d4). The blue arrows indicate the unexpected hole generated by CGAN.

Figure 6 depicts the brain MRI generated by the proposed method, Bicubic, and CGAN. Figures 6(b1)–(b5) specifically focus on the lesion regions, where both deep learning-based methods exhibit superior image resolution compared to Bicubic. In figure 6(b4), the synthetic image generated by CGAN displays spurious holes in the lesion region, indicated by the blue arrow, whereas the proposed method accurately generates a high-resolution image. Figure 6(d3) provides an absolute error map depicting the discrepancies between the ground truth and images generated by CGAN, highlighting substantial errors in the locations corresponding to the spurious holes. Figures 2(e1)–(e2) presents horizontal and oblique line profiles based on figure 6(c), revealing that CGAN consistently underestimates MRI intensities at multiple voxels, as indicated by the blue arrows, primarily due to the unexpected holes in the lesion region.

## 4. Discussion

With sharp increase of MRI applications in a wide range of diagnostic imaging and imaging-guided interventions, there are significant demands of high-resolution MR images to enhance detection and characterization of lesions for the precision of lesion and organ segmentation and quantitative measurement. However, the current imaging techniques for getting high-resolution images often come with trade-offs: longer acquisition times, which is challenging for clinic (Darestani *et al* 2021) including patient discomfort or pain, image degradation due to patient motion, and limited daily patients due to long wait times. One easy solution to expedite MRI acquisition is to decrease the image resolution along the phase encoding direction. However, this reduction in spatial detail can lead to problems of image quality, such as underestimating or overestimating lesion regions, which can subsequently impact the accuracy of auto-segmentation algorithms.

The proposed data-driven framework incorporates a diffusion probabilistic deep learning model, renowned for its ability to grasp data distributions, surpassing the semantic learning capabilities of traditional generative-adversarial networks. This data-driven approach stably harnesses the cutting-edge DDPM model to proficiently restore intricate local nuances and proficiently craft high-resolution MR images. The denoising procedure empowers the DDPM model to adeptly understand the comprehensive data distribution spanning various scales, encompassing global structures as well as local intricacies, facilitating the reconstruction of high-resolution details.

However, the DDPM model inherently incorporates randomness into denoising through a reverse Markov chain with ancestral sampling. When generating an image using DDPM, the model commences with a random noise vector and progressively denoises it to approximate the noise-free data distribution. At each denoising step, the model makes stochastic decisions that influence the noise vector’s evolution. This inherent randomness ensures that each sample produced by the model is distinct, even when utilizing the same initial noise vector. Figure 2 illustrates that the ENL values decrease as more samples are averaged by executing the DDPM multiple times. Figures 3(c1)–(c2) demonstrates a notable improvement in MAE (>10%) when averaging images from 3 to 5 samples. However, figures 3(c4)–(c6) reveals that the MAE plateaus around the value of 68. A similar trend can be observed in figure 4 using BraTS2020 datasets. To maintain the consistency of synthetic MR images, the proposed framework outlined in figure 1 defines criteria that synthetic MR images should have ENL values comparable to or lower than those of low-resolution images. Table 2 shows the optimal MSSIM and LPIPS that can be achieved by the proposed method. The proposed method can generate images that better agree with human expectations than other methods presented in this work. The proposed method can robustly achieve similar noise levels compared to the acquired MRI images. This feature is essential for radiotherapy since the accuracy of dose calculation in treatment planning is dominated by imaging-to-material characterization (Owrangi *et al* 2018, Chang *et al* 2022b). The material characterization method is machine-specific, and synthetic images should maximumly reproduce the features, such as noise level and perceptual similarities, to ensure the generated image set is applicable for radiotherapy.

**Figure 7.**
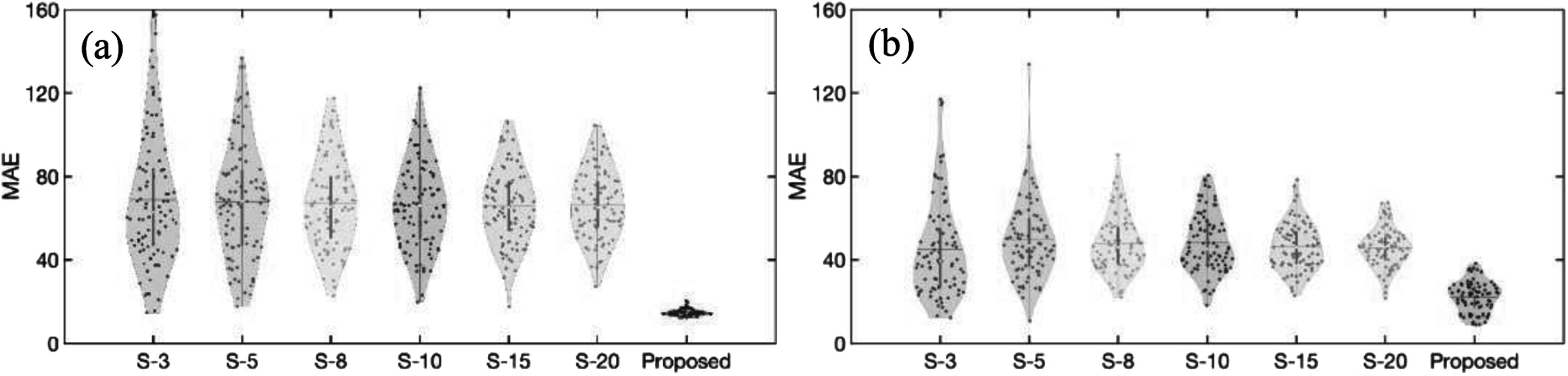
Comparisons of mean absolute error (MAE) distributions for all patients’ MR images from (a) the institutional prostate and (b) BraTS2020 datasets, including synthetic MR images by the proposed framework, and averaged MR images by running a DDPM multiple times to acquire different sample sizes (S-3 ∼ S-20).

Figure 7 illustrates that the proposed framework consistently achieves minimal MAE values, whether using institutional prostate data or the nonproprietary BraTS2020 dataset, compared to DDPM results obtained by averaging images from various samples. The results show that the proposed framework can minimize the randomness for image synthesis using DDPM models.

Figures 5(b3) and 6(b3) illustrate a common challenge encountered with traditional interpolation methods: the interpolated images tend to have blurred edges. Depending on the location of lesions, these blurred edges can be of critical significance, particularly in scenarios like brain tumor delineations where neighboring organs at risk, such as the optic nerve or chiasm, are involved. Conversely, figures 5(b5) and 6(b5) demonstrate that the proposed framework can generate high-resolution MR images without sacrificing the fine details of tissue edges. Tables 1 and 2 further substantiate this by confirming that the synthetic MR images produced by the proposed framework exhibit higher MSSIM values, indicative of superior image resolution compared to Bicubic. It is worth noting that image quality is not solely determined by Euclidean distances to ground truth; factors like resolution and spatial relationships also play a crucial role, which can be quantified through MSSIM. Although table 1 indicates that Bicubic achieves the optimal MAE, it still needs to improve overall image quality, as indicated by other evaluation metrics. Meanwhile, table 1 highlights that CGAN achieves the optimal MSSIM and LPIPS scores, but the associated *p*-values suggest no statistically significant difference compared to the performance of the proposed method. Figure 6(b5) shows that the proposed DDPM framework can stably generate high-resolution MRI without physically implausible low-intensity holes within the lesion region, which are absent in the ground truth. Ensuring stability is paramount in medical applications to prevent misdiagnosis or inappropriate treatment.

The proposed method has the potential to support the hyperspectral system (Li *et al* 2023), which integrates spatial and spectral information to achieve non-invasive diagnosis for bioinformatics. The system can analyze biological imaging samples in real time, but the high imaging rate usually compromise the resolution. A high-resolution image synthesis technique can enhance the hyperspectral system in dynamic processes for extracting molecular features from real-time biological imaging samples. Meanwhile, a high-resolution technique can reduce the uncertainty for auto-segmentation algorithms (Lenchik *et al* 2019) to accurate the dose distribution in radiotherapy treatment planning (Turcas *et al* 2023). The brain organs at risks, such as optic nerve and chiasm, usually involve fine structures that require high-resolution imaging to accurately evaluate the dosimetry impacts during radiotherapy (Alzahrani *et al* 2023).

The present data-driven framework incorporates denoising diffusion probabilistic deep learning to generate high-resolution synthetic MR images based on low-resolution inputs. Since the denoising process relies on a reverse Markov chain, the models perform inference step by step, gradually approaching noise-free images. This work’s computational aspect involves using an NVIDIA RTX A6000 GPU and a single run to generate 100 MR image slices for a patient in the super-inferior direction, typically taking approximately 5 h. While this may be acceptable for diagnostic purposes with no immediate urgency, the current inference time poses limitations on applications like adaptive radiotherapy and inter-fractional treatment verification. Future investigation will likely focus on accelerating diffusion probabilistic deep learning models to enhance the framework’s usability for potential clinical deployment. Two potential solutions for improving computational efficiency include optimizing the model architecture specifically for MRI and pre-computing the denoising process. The overarching objective of this work is to develop a data-driven framework capable of reliably synthesizing high-resolution images from low-resolution MRI inputs. This feasibility study has been demonstrated using institutional prostate and multi-institutional BraTS2020 datasets.

## 5. Conclusions

A data-driven framework has been demonstrated to synthesize high-resolution MR images by seamlessly integrating the diffusion probabilistic deep learning model. This integration generates superior quality and dependable images from low-resolution MRI inputs. Moreover, this innovative approach has the potential to substantially reduce the acquisition time required for MRI scans, thereby mitigating the occurrence of motion artifacts. Notably, the introduced method holds promise in providing high-resolution images, which can significantly enhance the accuracy of tasks like auto-segmentation, diagnosis, and radiotherapy in medical imaging.

## Data Availability

The data cannot be made publicly available upon publication because they contain sensitive personal information. The data that support the findings of this study are available upon reasonable request from the authors.

## Conflict of interest

The authors have no conflict of interest to disclose.

## Ethical statement

Emory IRB review board approval was obtained (IRB #114349), and informed consent was not required for this Health Insurance Portability and Accountability Act (HIPAA) compliant retrospective analysis. The research was conducted in accordance with the principles embodied in the Declaration of Helsinki and in accordance with local statutory requirements.
